# Acceptance of and hesitancy about COVID-19 vaccination among nursing students in clinical practice

**DOI:** 10.1371/journal.pone.0286640

**Published:** 2023-07-26

**Authors:** Saeryun Kim, Jisuk Lee, Hyunju Yang, Hyunkyun Kim

**Affiliations:** 1 College of Nursing, Chonnam National University, Gwangju, South Korea; 2 Chonnam National University Hwasun Hospital, Hwasun, Republic of Korea; Alexandria University High Institute of Public Health, EGYPT

## Abstract

**Background:**

Although vaccination of nursing students in clinical practice is important due to the possibility of COVID-19 infection and subsequent transmission to patients, some nursing students are hesitant to get vaccinated. Thus, it is necessary to identify the actual intentions and motivations of nursing students who have been vaccinated for COVID-19, even if their decisions were driven by clinical practice policy. The purpose of this study was to understand the nature of COVID-19 vaccine hesitancy among vaccinated nursing students in clinical practice and to examine their reasons for getting vaccinated despite such hesitancy.

**Method:**

A cross-sectional, descriptive study was conducted at two tertiary hospitals in South Korea from November 20, 2021, to December 17, 2021. The study recruited a convenience sample of 125 nursing students who were vaccinated for COVID-19. Data were analyzed using the chi-square test, Fisher’s exact test, and independent *t*-test.

**Results:**

Of the 125 nursing students, 51 (40.8%) reported vaccine hesitancy, among whom 88.2% reported that their hesitancy was due to the adverse effects and instability of the vaccine. It was also found that 70.6% of nursing students in the vaccine-hesitant group were eventually vaccinated due to clinical practice policy, whereas 67.6% of nursing students in the non-hesitant group were vaccinated to protect their health.

**Conclusions:**

COVID-19 vaccine hesitancy is prevalent among nursing students. Therefore, specific efforts should be made to provide education focusing on the safety, benefits, and efficacy of COVID-19 vaccines, implement mandatory vaccination policies for clinical practice, and give priority to vaccination opportunities to nursing students in order to reduce the hesitancy of nursing students to vaccines.

## Introduction

Vaccination is one of the most important measures to protect oneself from COVID-19, end the pandemic, and prevent the emergence of new variants [1]. COVID-19 vaccines were introduced in South Korea in February 2021. By May 2022, 86.8% of the population had reportedly received a second dose, and 64.7% of the population had received a third dose [2]. However, only 69.2% of the unvaccinated population was willing to receive the vaccine, leaving 30.8% of the population who was either hesitant or unwilling to be vaccinated [3]. People in their 20s, in particular, were identified as the least willing demographic in both domestic and international studies [4, 5].

Vaccine hesitancy is the unwillingness to receive a vaccine or the delay of vaccination despite full access to vaccination services and safe vaccines [6]. It is considered to be the definitive factor in the interruption of the successful control of the COVID-19 pandemic and a detriment to the achievement of herd immunity [7, 8]. According to a systematic review, the vaccine acceptance rate is less than 60% in many countries, including the United States, France, Italy, and Russia [9]. For healthcare workers (HCWs), vaccine acceptance rates range from 27.7–78.1% and are particularly low among nurses [9].

HCWs are at an increased risk of exposure to infectious diseases and pose a transmission risk to patients with deteriorated immunity. For this practical reason, HCWs are considered a top priority when it comes to vaccination. Nurses, in particular, have a higher risk of infectious disease morbidity because of their close proximity to patients during in-person clinical practice [10, 11]. As nursing students complete their clinical practice requirements in hospitals before they become nurses, they have a higher risk of acquiring infectious diseases [12]. This risk has led COVID-19 vaccine policies to prioritize nursing students as pre-HCWs.

Since most medical institutions recommend vaccination for frequent visitors because of infection control policies, nursing students preparing for clinical practice have to be vaccinated regardless of their willingness. Despite a high vaccination rate among nursing students in Korea, the proportion with a positive attitude toward vaccination has been shown to be very low at 29.6% [13]. The international situation is similar. In one study, only 43% of nursing students in seven European countries responded positively regarding COVID-19 vaccination [14]; elsewhere, 45.3% of nursing students in the United States were willing to be vaccinated [15]. This suggests that most nursing students do not have positive attitudes or intentions toward COVID-19 vaccination. Therefore, strategies to reduce vaccination hesitancy and build trust in vaccines will be important to combat COVID-19.

To date, there remains a limited understanding of COVID-19 vaccine hesitancy and the possible reasons for this attitude among nursing students. An appreciation of the actual intent and motivation of nursing students who have been vaccinated for COVID-19 will serve as a foundation for effective alternative strategies advising nursing students about vaccination in preparation for future pandemics. Thus, the purpose of this study was to understand the nature of COVID-19 vaccine hesitancy among vaccinated nursing students in clinical practice and to examine their reasons for getting vaccinated despite such hesitancy.

## Methods

### Study design and setting

This cross-sectional study was conducted at two tertiary hospitals managed by Chonnam National University in South Korea from November 20, 2021, to December 17, 2021. These two hospitals offer practicum opportunities in the fall semester (September 1 to February 28) and spring semester (March 1 to August 31). These two tertiary hospitals have made it mandatory to complete the second dose of the COVID-19 vaccine as a condition for nursing students to conduct clinical practice during the pandemic.

### Study population

All nursing students who completed clinical practice in the spring semester of 2021 (March 2021 to August 2021) were eligible for this study. There were 225 nursing students cross-vaccinated with AstraZeneca/Oxford, Moderna, and Pfizer/BioNTech vaccines prior to clinical practice.

### Sample size determination and sampling procedure

Under the study inclusion criteria, study participants had to be nursing students who had completed the first and second doses of the COVID-19 vaccine and be willing to participate in the study. Of 255 eligible students who were recruited, 140 students (54.9%) responded to the study survey, 15 did not report sufficient information in their survey responses and were excluded. Thus, 125 participants (49.0%) were selected for data analysis. A final sample size of *N* = 125 met the minimum calculated sample size (*n* = 116) considering the following assumptions: the proportion of COVID-19 vaccine hesitancy among students and trainees of healthcare professions (*p* = 18.9%) [16], the margin of error (*d* = 5%), and at the 95% confidence interval.

### Data collection

Ethical approval was obtained from the hospital’s institutional review board prior to initiation of this study. The list of 225 nursing students who completed clinical practice in the spring semester of 2021 was obtained from the administration departments of the participating hospitals. The list included only first and last names and email addresses. Data collection was conducted through an online survey via a Naver (a Korean website) form to minimize the risk of infection with COVID-19. Email invitations included a link to a website that included a short description of the study. Eligible students were directed to a webpage that contained the informed consent form. The participants took approximately 10 minutes to respond to the survey.

### Study instruments

#### Structured questionnaires

The items of the structured questionnaire were constructed through a review of relevant literature. The questionnaires consisted of general characteristics, COVID-19 exposure–related characteristics, COVID-19 vaccination–related characteristics, and six questions about preventive health behavior against COVID-19. The items about general characteristics, COVID-19 exposure-related characteristics, and COVID-19 vaccination-related characteristics were selected through a literature review that identified nursing students’ intentions for influenza vaccination and COVID-19 vaccination [17–19]. Questions regarding preventive health behaviors against COVID-19 were extracted from the COVID-19 Prevention Guidelines of the Korea Centers for Disease Control and Prevention Agency (KDCA) [20].

*General characteristics*. General characteristics were measured using six questions. These included (1) sex, (2) age (*20*, *21*, *22*, *23*, *24 years*, *or above*), (3) grade level (*junior or senior*), (4) perceived health status (*very good*, *good*, *fair*, *poor*, *or very poor*), (5) underlying medical conditions (*yes or no*), and (6) influenza vaccination history (*annual*, *sometimes*, *or never*). Responses regarding perceived health status were grouped into two levels: poor (*very poor*, *poor*, *or fair*) and good (*very good or good*). Responses regarding influenza vaccination history were grouped into two levels: annual (*annual*) and sometimes or never (*sometimes or never*).

*COVID-19 exposure–related characteristics*. COVID-19 exposure–related characteristics were measured using three items: (1) experience of contact with confirmed cases (*yes or no*), (2) quarantine experience post-contact with confirmed cases (*yes or no*), and (3) confirmed cases among family members or friends (*yes or no*).

*COVID-19 vaccination–related characteristics*. COVID-19 vaccination–related characteristics were measured using 15 items. As the first item, COVID-19 vaccine hesitancy was measured with “Have you been hesitant to get vaccinated against COVID-19?” This question had binary response options of *yes or no*. Item 2 was about the reason for the hesitation to get vaccinated. Only participants who responded ‘*yes*’ to the first question were asked, “Why did you hesitate to get vaccinated against COVID-19?” Participants could choose one or more of these responses: “concerns about the safety of the COVID-19 vaccine,” “distrust of the vaccine’s effectiveness,” “low awareness of COVID-19’s health risks,” “fear of needles and injections,” “difficulty taking time off from school to get the vaccine,” and “other.” Item 3 was about the reasons for getting vaccinated against COVID-19. Participants were asked, “Why did you get vaccinated against COVID-19?” Participants could choose one or more of these responses: “clinical practice policy,” “protection of own health,” “provision of the opportunity for vaccination,” “responsibility as a pre-healthcare worker,” “concerns about the spread of infection,” “cost-free,” “benefit of quarantine pass,” “recommendation of parents,” and “other.” Items 4 and 5 measured types of COVID-19 vaccine with three options: AstraZeneca/Oxford, Moderna, and Pfizer/BioNTech. Items 6 and 7 measured the adverse effects of COVID-19 vaccines as *yes or no*. Items 8 and 9 were about the types of adverse effects (*fever*, *muscle aches*, *headache*, *nausea/vomiting*, *diarrhea*, *fatigue*, *allergic reactions*, *pain/redness/swelling at the injection site*, *or other*). Participants were allowed to choose multiple responses. Items 10 and 11 measured the severity of the adverse effects on a 10-point scale (*0 = not severe at all*, *10 = extremely severe*). Items 12 and 13 measured the number of days of adverse effects. To respond to items 4 through 13, participants responded about the first and second doses of the COVID-19 vaccine they received separately. For item 14, the intention to recommend COVID-19 vaccination to family members or friends was indicated as *yes or no*. A second part of this item was about the reason for the recommendation of COVID-19 vaccination to family members or friends. Participants could choose one or more of these responses: “to end the COVID-19 pandemic,” “to alleviate anxiety toward COVID-19,” “to avoid further aggravation of the COVID-19 pandemic due to mutated variant,” “for ease of compliance with COVID-19 quarantine guidelines,” “trust in the vaccine’s efficacy,” “absence of serious adverse effects after vaccination,” and “other.”

*Preventive health behaviors against COVID-19*. Preventive health behaviors against COVID-19 were measured using six items. Participants responded using a 4-point Likert scale (*1 = hardly ever*, *2 = some of the time*, *3 = most of the time*, *and 4 = all the time*). The total score ranged from 6 to 24, and higher scores were considered to be better for preventive health activities. Responses were grouped into two levels: activities not performed (*hardly ever* and *some of the time*) and activities performed (*most of the time* and *all the time*) (S1 File).

#### COVID-19–related knowledge

COVID-19–related knowledge was measured using a questionnaire modified by Kim [21]. The questionnaire consists of 20 items with the binary response options of *true* or *false*. One point is given for a correct answer, and zero points are given for incorrect answers. The total score ranges from 0 to 20, with a higher score indicating a greater level of knowledge. The content validity index (CVI) of this questionnaire was 0.98.

### Data analysis

The collected data were analyzed using SPSS Statistics for Windows, version 26 (IBM Corp. Armonk, NY, USA), with a two-sided significance level of α = 0.05. Categorical data were analyzed using frequencies and percentages, and continuous data were analyzed using means and standard deviations. The chi-square test, Fisher’s exact test, and the independent t-test were used to examine differences in general characteristics and in those pertaining to COVID-19, including preventive health behaviors, knowledge, and vaccination-related characteristics.

### Ethical considerations

This study was reviewed and approved by the research ethics committee of Chonnam National University Hwasun Hospital (IRB No.: CNUHH-2021-213). Information about the aim of the study, participant anonymity, and voluntary participation was provided to the participants, and written informed consent was obtained from the participants.

## Results

### Vaccine hesitancy and reasons

**Fig 1:**
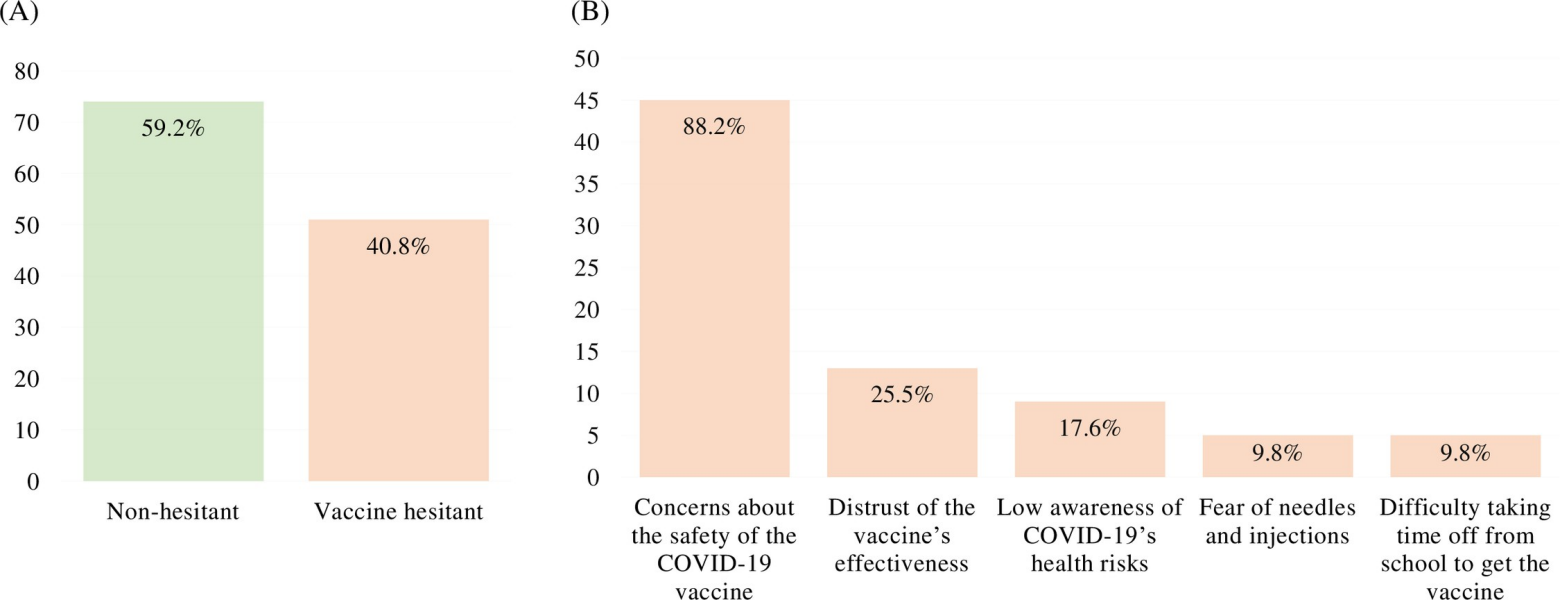
Reasons for COVID-19 vaccine hesitancy among nursing students (multiple responses permitted). (A) Prevalence of vaccine hesitancy among nursing students. (B) Reasons why nursing students in the vaccine-hesitant group hesitated to undergo COVID-19 vaccination.

Regarding COVID-19 vaccine hesitancy, 40.8% of the 125 participants (*n* = 51) were hesitant, and 59.2% (*n* = 74) were not hesitant. Among the nursing students who hesitated to get the COVID-19 vaccine, 88.2% (*n* = 45) were concerned about the safety of the COVID-19 vaccine, including adverse effects. In addition, distrust of the vaccine’s effectiveness (25.5%), low awareness of COVID-19’s health risks (17.6%), fear of needles and injections (9.8%), and difficulty taking time off from school to get the vaccine (9.8%) were other indicated reasons (Fig 1).

### General and COVID-19 exposure–related characteristics

Regarding general characteristics, 88.8% of the 125 students (*n* = 111) were female, 66.4% were younger than 22 years of age, and 60.8% were seniors in college. There were no statistically significant differences in perceived health status and chronic disease between the vaccine-hesitant group and the non-hesitant group. In the vaccine-hesitant group, 64.7% of participants received the influenza vaccine annually, compared with 54.1% of the non-hesitant group; however, there were no statistically significant differences. As for the COVID-19 exposure-related characteristics, experience of contact with confirmed cases, quarantine experience after such contact, or a history of confirmed cases among family members or friends, showed no statistically significant differences between the two groups (Table 1).

**Table 1:** Comparison of general characteristics and COVID-19 exposure–related characteristics between the vaccine-hesitant group and the vaccine non-hesitant group.

Variables	Total	Vaccine hesitant	Non-hesitant	χ^2^	*p*
	(n = 125)	(n = 51)	(n = 74)		
	n (%)				
**General characteristics**					
Sex					
Male	14 (11.2)	6 (11.8)	8 (10.8)	0.28	> 0.999
Female	111 (88.8)	45 (88.2)	66 (89.2)		
Age					
≤ 22 years	83 (66.4)	35 (68.6)	48 (64.9)	0.19	0.704
>22 years	42 (33.6)	16 (31.4)	26 (35.1)		
Grade level					
Junior	49 (39.2)	23 (45.1)	26 (35.1)	1.26	0.271
Senior	76 (60.8)	28 (54.9)	48 (64.9)		
Perceived health status					
Poor	33 (26.4)	15 (29.4)	18 (24.3)	0.40	> 0.999
Good	92 (73.6)	36 (70.6)	56 (75.7)		
Chronic disease					
No	113 (90.4)	46 (90.2)	67 (90.5)	0.01	> 0.999
Yes	12 (9.6)	5 (9.8)	7 (9.5)		
Received the seasonal influenza vaccine					
Sometimes or never	52 (41.6)	18 (35.3)	34 (45.9)	1.41	0.271
Annual	73 (58.4)	33 (64.7)	40 (54.1)		
**COVID-19 exposure-related characteristics**					
Experience of contact with confirmed cases					
No	98 (78.4)	40 (78.4)	58 (78.4)	0.01	> 0.999
Yes	27 (21.6)	11 (21.6)	16 (21.6)		
Quarantine experience post-contact with confirmed cases					
No	111 (88.8)	43 (84.3)	68 (91.9)	1.74	0.250
Yes	14 (11.2)	8 (15.7)	6 (8.1)		
Confirmed cases among family members or friends					
No	100 (80.0)	38 (74.5)	62 (83.8)	1.62	0.256
Yes	25 (20.0)	13 (25.5)	12 (16.2)		

### Preventive health behaviors and knowledge of COVID-19

Engaging in preventive health behaviors for COVID-19, including mask-wearing, handwashing, showering after outdoor activities, refraining from eating out or going outside, and avoiding public facilities and confined spaces, showed no statistically significant differences between the two groups. As for the COVID-19 knowledge, there were no significant differences between the two groups (Table 2).

**Table 2:** Comparison of preventive health behaviors and knowledge of COVID-19 between the vaccine-hesitant group and the vaccine non-hesitant group.

Variables	Total	Vaccine hesitant	Non-hesitant	t or χ^2^	*p*
	(n = 125)	(n = 51)	(n = 74)		
	M ± SD or n (%)				
**Preventive health behaviors during the COVID-19 pandemic** ^**a**^					
Mask-wearing in the right way					
Some of the time or hardly ever	0 (0.0)	0 (0.0)	0 (0.0)	0.00	1.000
All or most of the time	125 (100.0)	51 (100.0)	74 (100.0)		
Handwashing					
Some of the time or hardly ever	1 (0.8)	1 (2.0)	0 (0.0)	1.46	0.408
All or most of the time	124 (99.2)	50 (98.0)	74 (100.0)		
Showering after outdoor activities					
Some of the time or hardly ever	12 (9.6)	4 (7.8)	8 (10.8)	0.31	0.760
All or most of the time	113 (90.4)	47 (92.2)	66 (89.2)		
Refraining from eating out or going outside					
Some of the time or hardly ever	34 (27.2)	14 (27.5)	20 (27.0)	0.01	0.958
All or most of the time	91 (72.8)	37 (72.5)	54 (73.0)		
Avoiding public facilities					
Some of the time or hardly ever	37 (29.6)	16 (31.4)	21 (28.4)	0.13	0.842
All or most of the time	88 (70.4)	35 (68.6)	53 (71.6)		
Avoiding confined spaces					
Some of the time or hardly ever	17 (13.6)	9 (17.6)	8 (10.8)	1.20	0.298
All or most of the time	108 (86.4)	42 (82.4)	66 (89.2)		
**Knowledge of COVID-19** ^**b**^	15.99 ± 2.07	15.86 ± 2.36	16.08 ± 1.86	0.58	0.565

^a^ Items based on the COVID-19 Prevention Guidelines of the Korea Centers for Disease Control and Prevention Agency (KDCA) [20]

^b^ Measured by the knowledge tool modified by Kim et al [21]

### Reasons for receiving COVID-19 vaccination

**Fig 2:**
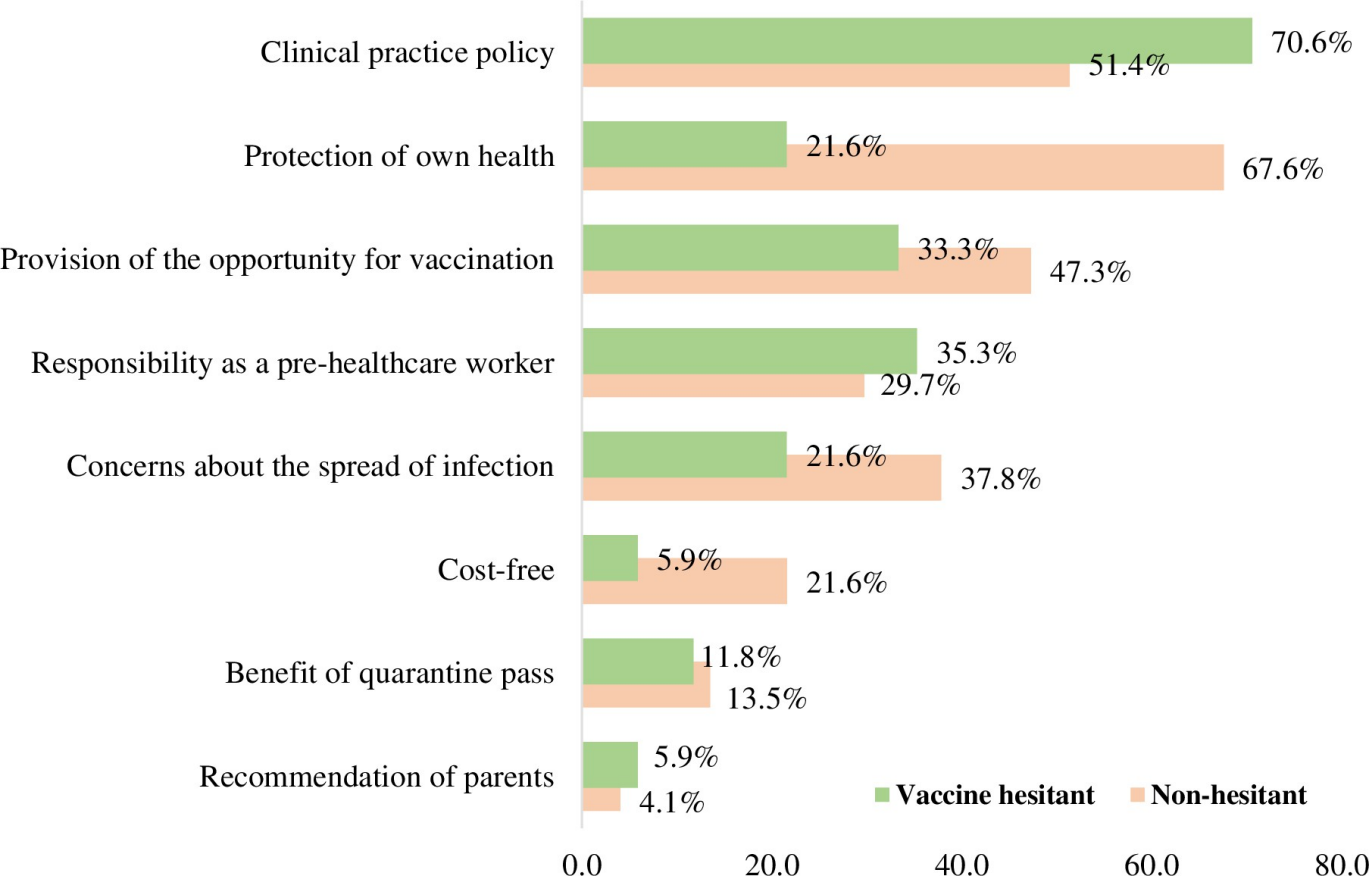
Reasons for deciding to undergo COVID-19 vaccination (multiple responses permitted).

Nursing students in the vaccine-hesitant group reported their reasons for getting vaccinated against COVID-19 as clinical practice policy (70.6%), followed by responsibility as a pre-HCW (35.3%), provision of the opportunity for vaccination (33.3%), protection of own health (21.6%), and concerns about the spread of infection (21.6%). On the other hand, nursing students in the non-hesitant group responded that their reasons for receiving COVID-19 vaccination were protection of own health (67.6%), followed by clinical practice policy (51.4%), provision of the opportunity for vaccination (47.3%), concerns about the spread of infection (37.8%), and responsibility as a pre-HCW (29.7%) (Fig 2).

### COVID-19 vaccination–related characteristics: First and second doses

In both groups, the Moderna vaccine was the most frequently received for the first and second COVID-19 vaccine doses, and there was no statistically significant difference in vaccination hesitancy according to the type of vaccine. There were no statistically significant differences between the two groups in the experiences of adverse effects, the types of adverse effects, the severity of adverse effects, or the durations of adverse effects associated with the first dose of the COVID-19 vaccine. Regarding the second dose of the COVID-19 vaccine, there were no statistically significant differences between the two groups in terms of the experience of adverse effects, the severity of adverse effects, or the duration of adverse effects. However, 76.7% of nursing students in the vaccine-hesitant group (*n* = 23) reported fatigue, whereas 46.7% of the non-hesitant group (*n* = 21) had fatigue, which was a statistically significant difference (χ^2^ = 6.68, *p* = 0.010) (Table 3).

**Table 3:** Comparison of COVID-19 vaccination-related characteristics between the vaccine-hesitant group and the vaccine non-hesitant group.

Variables	Total	Vaccine hesitant	Non-hesitant	t or χ^2^	*p*
	(n = 125)	(n = 51)	(n = 74)		
	M ± SD or n (%)				
***COVID-19 vaccine*: *first dose***					
Types of vaccine					
AstraZeneca	53 (42.4)	24 (47.1)	29 (39.2)	0.79	0.674
Moderna	69 (55.2)	26 (51.0)	43 (58.1)		
Pfizer	3 (2.4)	1 (2.0)	2 (2.7)		
Adverse effects of the COVID-19 vaccine					
No	38 (30.4)	14 (27.5)	24 (32.4)	0.35	0.562
Yes	87 (69.6)	37 (72.5)	50 (67.6)		
Types of adverse effects of the COVID-19 vaccine (*n* = 87)*					
Fever	39 (44.8)	17 (45.9)	22 (44.0)	0.03	0.857
Muscle aches	75 (86.2)	33 (89.2)	42 (84.0)	0.48	0.488
Headache	41 (47.1)	19 (51.4)	22 (44.0)	0.46	0.497
Nausea/vomiting^#^	11 (12.6)	4 (10.8)	7 (14.0)	-	0.753
Diarrhea*	2 (2.3)	2 (5.4)	0 (0.0)	-	0.178
Fatigue	42 (48.3)	20 (54.1)	22 (44.0)	0.86	0.354
Allergic reactions^#^	2 (2.3)	1 (2.7)	1 (2.0)	-	0.999
Pain/redness/swelling at the injection site	31 (35.6)	17 (45.9)	14 (28.0)	2.99	0.084
Severity of adverse effects	3.42 ± 2.83	3.98 ± 2.93	3.04 ± 2.72	1.84	0.068
Duration of adverse effects (days)	1.81 ± 1.42	2.08 ± 1.50	1.62 ± 1.34	1.79	0.077
***COVID-19 vaccine*: *second dose***					
Types of vaccine					
AstraZeneca	52 (41.6)	24 (47.1)	28 (37.8)	1.31	0.520
Moderna	69 (55.2)	26 (51.0)	43 (58.1)		
Pfizer	4 (3.2)	1 (2.0)	3 (4.1)		
Adverse effects of the COVID-19 vaccine					
No	50 (40.0)	21 (41.2)	29 (39.2)	0.05	0.854
Yes	75 (60.0)	30 (58.8)	45 (60.8)		
Types of adverse effects of the COVID-19 vaccine (*n* = 60)*					
Fever	44 (58.7)	18 (60.0)	26 (57.8)	0.04	0.848
Muscle aches	59 (78.7)	25 (83.3)	34 (75.6)	0.65	0.421
Headache	44 (58.7)	18 (60.0)	26 (57.8)	0.04	0.848
Nausea/vomiting^#^	11 (14.7)	3 (10.0)	8 (17.8)	-	0.509
Diarrhea^#^	2 (2.7)	0 (0.0)	2 (4.4)	-	0.514
Fatigue	44 (58.7)	23 (76.7)	21 (46.7)	6.68	0.010
Allergic reactions^#^	4 (5.3)	2 (6.7)	2 (4.4)	-	0.999
Pain/redness/swelling at the injection site	28 (37.3)	12 (40.0)	16 (35.6)	0.15	0.697
Severity of adverse effects	3.23 ± 3.20	3.31 ± 3.29	3.18 ± 3.16	0.24	0.816
Duration of adverse effects (days)	1.43 ± 1.48	1.53 ± 1.54	1.36 ± 1.44	0.61	0.543

* Multiple responses permitted

^#^ Fisher’s exact test

### Recommendation and reasons for recommending COVID-19 vaccination

There were statistically significant differences between the two groups regarding the intention to recommend COVID-19 vaccination to family members or friends, with only 49.0% of the vaccine-hesitant group (*n* = 24) intending to recommend vaccination, compared with 74.3% (*n* = 55) of the non-hesitant group (χ^2^ = 8.39, *p* = 0.005). Nursing students in the vaccine-hesitant group reported the following top reasons for recommending COVID-19 vaccination: to end the COVID-19 pandemic (91.7%), followed by relief of COVID-19 anxiety (58.3%). Similarly, nursing students in the non-hesitant group reported the following top reasons for recommending COVID-19 vaccination: to end the COVID-19 pandemic (67.3%), followed by relief of COVID-19 anxiety (60.0%) (Table 4). None of the nursing students in the vaccine-hesitant group trusted the efficacy of the COVID-19 vaccine, but 40.0% of the nursing students in the non-hesitant group (*n* = 22) trusted the efficacy of the COVID-19 vaccine (χ^2^ = 16.07, *p* = <0.001).

**Table 4:** Recommendation and reasons for recommending COVID-19 vaccination between the vaccine-hesitant group and the vaccine non-hesitant group.

Variables	Total	Vaccine hesitant	Non-hesitant	χ^2^	*p*
	(n = 125)	(n = 51)	(n = 74)		
	n (%)				
**Intention to recommend vaccination against COVID-19 to others**					
Not recommended	45 (46.0)	26 (51.0)	19 (25.7)	8.39	0.005
Recommended	80 (64.0)	24 (49.0)	55 (74.3)		
**Reasons for recommending vaccination** (*n* = 80)*					
To end the COVID-19 pandemic	59 (73.8)	22 (91.7)	37 (67.3)	0.53	0.469
To alleviate anxiety toward COVID-19	47 (58.8)	14 (58.3)	33 (60.0)	0.01	0.949
To avoid further aggravation of the COVID-19 pandemic due to mutated variant	29 (36.3)	9 (37.5)	20 (36.4)	1.69	0.194
For ease of compliance with COVID-19 quarantine guidelines	25 (31.3)	9 (37.5)	16 (29.1)	0.03	0.856
Trust in the vaccine’s efficacy	22 (27.5)	0 (0.0)	22 (40.0)	16.07	<0.001
Absence of serious adverse effects after vaccination	21 (26.3)	9 (37.5)	12 (21.8)	0.45	0.501

* Multiple responses permitted

## Discussion

The study participants were nursing students who had completed second doses of COVID-19 vaccination for clinical practice, and the study found that 40.8% of nursing students were hesitant to get vaccinated. The proportion of nursing students with positive attitudes toward COVID-19 vaccination ranges from 29.6% to 50.9% domestically and overseas [13–15, 22]. A study of HCWs in Saudi Arabia, where 90% had already received second COVID-19 vaccine doses, found that the mean score for unwillingness to undergo vaccination was 2.71 out of 5 [23]. Taken together, these findings imply that not all vaccinated people are willing to be vaccinated or have positive attitudes about vaccination.

Vaccine hesitancy among HCWs could affect not only public hesitancy toward COVID-19 vaccination but also the immunity of high-risk groups, among whom morbidity causes severe symptoms [24]. Nursing students in clinical practice have as high a risk of infection as medical practitioners do, resulting in strong advice that these pre-HCWs get vaccinated. As pre-HCWs, their attitudes toward vaccination may affect public vaccination rates because nursing students serve as health educators in local communities [25, 26]. Nursing students who show an unwillingness to receive vaccines are likely to have negative attitudes toward promoting vaccination to the public. Therefore, efforts to inculcate positive attitudes about vaccines should be prioritized to increase vaccination rates among nursing students.

This study found that concerns about vaccine safety and adverse effects led to nursing students’ hesitancy to receive vaccines, which is consistent with findings from previous studies in which 81.0% of nursing students [22] and 73% of health and medical science students [27] were reluctant to undergo vaccination for the same reasons. In a recent systematic review, vaccine instability was the strongest determinant of HCWs vaccine hesitancy. More specifically, the fast speed of vaccine development and approval, as well as the possibility of long-term adverse effects, have contributed to vaccine hesitancy among HCWs [28]. This finding suggests that an effective approach to providing evidence-based information on the efficacy and adverse effects of vaccines, rather than a mere emphasis of their importance, is needed to promote vaccination. To inspire nursing students to have positive attitudes toward vaccines, educational institutions should provide vaccination education customized for pre-HCWs rather than simply imposing duties and responsibilities on students as future medical professionals.

Another important finding in this study was that the group who hesitated to undergo vaccination had more fatigue associated with the second vaccination dose (S2 File). Although the vaccine itself may have caused fatigue, it is also possible that the nocebo effect, in which symptoms can be amplified by individual negative expectations and related factors, may have contributed [29]. Rief [30] noted that responsiveness to COVID-19 vaccines and vaccine hesitancy can be affected by psychosocial factors, especially the nocebo effect. In addition, it is important to have a positive attitude toward the vaccine because negative expectations and attitudes toward vaccination, such as vaccine hesitancy and concerns about adverse effects, can exacerbate physical adverse effects [31].

In this study, about 70% of the nursing students reported that they eventually underwent vaccination despite not wanting to because of clinical practice obligations. This suggests that they were vaccinated to follow clinical practice policies that were contrary to individual beliefs and will. Strategies for maximizing vaccination rates among nursing students may include the development of guidelines for obligatory vaccination. However, nursing students in the non-hesitant group voluntarily underwent vaccination to protect their own health instead of relying on passive vaccination. In other words, positive attitudes toward vaccination could eventually lead to vaccination. Strategies including education on the safety, benefits, and efficacy of COVID-19 vaccines, as well as psychosocial programs that reduce the nocebo effect, may be effective in strengthening positive attitudes toward vaccination.

In this study, 64.0% of nursing students who were vaccinated against COVID-19 reported that they were willing to recommend COVID-19 vaccination to family members and friends. On the other hand, 44.0% of medical and health science students who had not been vaccinated against COVID-19 indicated that they were willing to give advice to others on COVID-19 vaccination [24]. The relatively high likelihood of recommending vaccination to others in this study can be explained by the fact that anxiety about COVID-19 was relieved after actual vaccination. Even in the hesitant group in this study, 91.7% of nursing students reported that they would recommend vaccination to end the COVID-19 pandemic, suggesting an acknowledgement of the role of herd immunity in ending the pandemic and a realization that advising others would help achieve this goal. A positive attitude toward public health can be a determinant of nursing students getting vaccinated [32]. Therefore, the importance of herd immunity should also be emphasized in order to increase vaccination rates.

Regardless of their intention to undergo vaccination, nursing students in this study followed precautionary measures against COVID-19, such as mask-wearing and handwashing, which is believed to be due to the government’s strong policies and active publicity on COVID-19 prevention. However, in this study, there was no significant relationship between COVID-19–related knowledge and vaccination intention, which was consistent with the findings of previous studies on clinical nurses [33]. From these results, it can be inferred that knowledge about disease does not directly affect vaccination intention; in contrast, vaccine-related factors affect vaccination intention. A study by Manning [15] found that nursing students had not only low intention to immunize but also low knowledge about vaccine development. These findings suggest that interventions to reduce vaccine refusal and provide evidence-based education can increase vaccination intention. Therefore, providing nursing students sufficient opportunities to learn about vaccines at school would be considered an effective approach to increasing their vaccination intention.

### Study limitations

We acknowledge some limitations of our study. First, the limited sample size focusing only on nursing students in clinical practice at two tertiary hospitals limits the generalizability of the study findings. Thus, larger-scale multicenter research is necessary. Second, unlike previous studies where participants were examined prior to vaccination, this study focused on post-vaccination participants whose vaccination experiences may have changed their attitudes or intentions. Therefore, the rapid and lasting impact of the COVID-19 pandemic and fluctuations in influence may have also impacted nursing students’ intentions or motivations to get vaccinated. Third, to minimize missing values, which are considered a limitation of online surveys, the survey items (e.g., age) were configured in categorical formats, and because of this, there were limitations in presenting the measured values of some variables as means.

### Study implications

Previous studies have focused primarily on identifying possible reasons for vaccination intentions prior to vaccination in nursing students. However, in this study, the approach that facilitated the identification of the actual reasons why nursing students got vaccinated despite their unwillingness will help improve our understanding of the possible causes of vaccination hesitancy and how to overcome low vaccination rates. The findings of this study will contribute to the development of strategies to improve vaccination rates among nursing students and strengthen their positive attitudes toward vaccines in preparation for the next epidemic.

## Conclusion

In this study, despite the fact that nursing students were vaccinated for clinical practice in the context of the COVID-19 pandemic, about 41% of them were hesitant to get vaccinated. It is necessary to establish the safety of vaccines and provide sufficient vaccine-related safety education before vaccination to minimize vaccine hesitancy among nursing students. In addition, as a practical effort, it is necessary to strengthen mandatory vaccination requirements for clinical practice and prioritize vaccination opportunities for nursing students. To achieve this, governments, educational institutions, and medical institutions should collaborate and cooperate.

## Supporting information

S1 File Preventive health behaviors to prevent the spread of COVID-19. (DOCX)

S2 File Logistic regression analysis of risk factors for vaccine hesitancy among nursing students. (DOCX)
