# Supplementary material for: Acceptance of and hesitancy about COVID-19 vaccination among nursing students in clinical practice
Source: PLoS One. 2023 Jul 26;18(7):e0286640. doi: 10.1371/journal.pone.0286640 (PMC10370686; doi:10.1371/journal.pone.0286640)
Supplement: S1 File — (DOCX) [file pone.0286640.s001.docx]

The following is about preventive health behaviors to prevent the spread of COVID-19. Please mark the average level of preventive health behavior below for the past month.

**Table S1.** Preventive health behaviors to prevent the spread of COVID-19

| **Question** | **Hardly ever** | **Some of the time** | **Most of the time** | **All of time** |
| --- | --- | --- | --- | --- |
| 1. Mask-wearing in the right way |  |  |  |  |
| 2. Handwashing |  |  |  |  |
| 3. Showering after outdoor activities |  |  |  |  |
| 4. Refraining from eating out or going outside |  |  |  |  |
| 5. Avoiding public facilities (e.g. Restaurants, cafes, indoor sports facilities, movie theaters, shopping malls, and public baths, etc.) |  |  |  |  |
| 6. Avoiding confined spaces (e.g. Karaoke, PC cafes, etc.) |  |  |  |  |

Items based on the COVID‑19 Prevention Guidelines of the Korea Centers for Disease Control and Prevention Agency (KDCA) [20]
