# Supplementary material for: Acceptance of and hesitancy about COVID-19 vaccination among nursing students in clinical practice
Source: PLoS One. 2023 Jul 26;18(7):e0286640. doi: 10.1371/journal.pone.0286640 (PMC10370686; doi:10.1371/journal.pone.0286640)
Supplement: S2 File — (DOCX) [file pone.0286640.s002.docx]

**Data analysis**

Risk factors of vaccine hesitancy were determined by binary logistic regression analysis using all available variables of patients' general characteristics, COVID-19 exposure-related characteristics, COVID-19 vaccination-related characteristics, preventive health behaviors against COVID-19, and COVID-19-related knowledge. Variables with p-values less than 0.1 identified in univariate analysis were carried on to a backward stepwise logistic regression to determine risk factors for vaccine hesitancy. Adjusted odds ratios (AORs) were determined for each variable, and a p-value less than 0.05 was considered statistically significant.

**Results**

Univariate analysis suggested that pain/redness/swelling at the injection site (after first dose of COVID-19 vaccine), severity of adverse effects (after first dose of COVID-19 vaccine), duration of adverse effects (after first dose of COVID-19 vaccine), and fatigue (after second dose of COVID-19 vaccine) were associated with increased risk of vaccine hesitancy among nursing students. Logistic regression analysis confirmed that fatigue after the second dose of COVID-19 vaccine was significantly associated with a 3.8-fold increase in the odds of vaccine hesitancy (OR: 3.835; 95% confidence interval [CI]: 1.345–10.933, *p* = 0.012).

**Table S2.** Logistic regression analysis of risk factors for vaccine hesitancy among nursing students

| **Variables** |  | **OR (95% CI)** | ***p*** |
| --- | --- | --- | --- |
| Fatigue (second dose) | Yes | 3.835(1.345–10.933) | 0.012 |
|  | No | 1 |  |
| Pain/redness/swelling at the injection site (first dose) | Yes | 1.137(0.407–3.182) | 0.806 |
|  | No | 1 |  |
| Severity of adverse effects (first dose) |  | 1.157(0.903–1.484) | 0.249 |
| Duration of adverse effects (first dose) |  | 0.827(0.498–1.371) | 0.461 |

Note: OR; Odds Ratio, 1 = reference
